# Optimization of medium composition for in vitro shoot proliferation and growth of date palm cv. Mejhoul

**DOI:** 10.1007/s13205-016-0430-x

**Published:** 2016-05-12

**Authors:** Mouaad Amine Mazri, Reda Meziani, Jamal El Fadile, Az-eddine Ezzinbi

**Affiliations:** 1 Institut National de la Recherche Agronomique, CRRA-Marrakech, UR Agro-Biotechnologie, Laboratoire de Biotechnologie Végétale, Avenue Mohammed 6, B.P. 533, Marrakech, Morocco; 2 Institut National de la Recherche Agronomique, CRRA-Errachidia, UR Systèmes Oasiens, Laboratoire National de Culture des Tissus du Palmier Dattier, Avenue Moulay Ali Cherif, B.P. 2, Errachidia, Morocco

**Keywords:** Carbon source, l-Glutamine, Mineral salts, Myo-inositol, Organogenesis, *Phoenix dactylifera* L.

## Abstract

The effects of major mineral salts, l-glutamine, myo-inositol and carbon source on shoot bud proliferation of date palm (*Phoenix dactylifera* L.) cv. Mejhoul were evaluated. Different concentrations of ammonium nitrate (NH_4_NO_3_; 550, 825 or 1650 mg/L), potassium nitrate (KNO_3_; 633.3, 950 or 1900 mg/L), calcium chloride dehydrate (CaCl_2_·2H_2_O; 147, 220 or 440 mg/L), potassium dihydrogen phosphate (KH_2_PO_4_; 57, 85 or 170 mg/L), magnesium sulfate heptahydrate (MgSO_4_·7H_2_O; 123, 185 or 370 mg/L), l-glutamine and myo-inositol (0.5, 1, 1.5 and 2 g/L), sucrose, sorbitol, mannitol or commercial granulated sugar (10, 20, 30, 40 or 50 g/L) were tested. The highest number of shoot buds per explant (18.7) occurred on the medium containing 825 mg/L NH_4_NO_3_, 1900 mg/L KNO_3_, 220 mg/L CaCl_2_·2H_2_O, 170 mg/L KH_2_PO_4_, 370 mg/L MgSO_4_·7H_2_O as well as 1 g/L l-glutamine, 2 g/L myo-inositol and 30 g/L sucrose. The results showed that the frequency of hyperhydricity significantly increased in media containing 1650 mg/L NH_4_NO_3_. The concentrations of l-glutamine, myo-inositol and carbon source significantly affected the number of shoot buds per explant. However, they had no effect on hyperhydricity, tissue browning and precocious rooting. Shoots of 4.5–6.0 cm in length were isolated and transferred onto hormone-free media for elongation and rooting. After 3 months, the developed plantlets were successfully transplanted in a glasshouse and over 90 % survived acclimatization.

## Introduction

Date palm (*Phoenix dactylifera* L.) is an agronomically, socioeconomically and ecologically important crop species in the Middle East and North Africa. The worldwide population of date palm trees is estimated to be around 150 million (Al-Khayri et al. 2015). In Morocco, date palm covers 50,000 ha with 5.4 million trees and 453 cultivars (Sedra 2015). Among these cultivars, cv. Mejhoul, also known as Medjool, Medjhool, Medjehuel and Mejhul (Elhoumaizi et al. 2006), is the most sought after and most famous cultivar worldwide (Sedra 2015). Mejhoul is also the most popular and most desired variety by the Moroccan farmers and consumers. Unfortunately, cv. Mejhoul is threatened by bayoud, a very dangerous disease caused by the fungus *Fusarium oxysporum* f. sp. *albedinis*, which has decimated over 12 million palm trees (Saker 2011), and significantly decreased the population of cv. Mejhoul (Sedra 2011).

To preserve cv. Mejhoul from bayoud and to produce enough plants to satisfy the high demand of farmers and consumers, developing a large-scale propagation system then planting regenerants in bayoud-free areas is the only practical solution. Along this line, a micropropagation program through organogenesis was released in 2010 by the Moroccan Ministry of Agriculture, including cultivars of high fruit quality such as Najda, Boufeggous and Bouskri. The amount of plants of cv. Mejhoul aimed to be produced annually within this program is 67 % of the total amount of plants produced. However, micropropagation of date palm is genotype dependent (Jain 2012), and cv. Mejhoul is one of the most recalcitrant cultivars to in vitro manipulations. Therefore, developing an efficient regeneration system through organogenesis for this cultivar would require a high optimization of media components and culture conditions.

Studies on date palm micropropagation through organogenesis are scarce. In the past few years, regeneration systems through this technique were reported in some cultivars; e.g., cv. Maktoom (Khierallah and Bader 2007), cv. Dhakki (Khan and Bi Bi 2012), cv. Hillawi (Al-Mayahi 2014) and cv. Boufeggous (Mazri 2015). However, the majority of works focused on the effects of plant growth regulators (PGRs). The effects of other factors such as mineral salts, l-glutamine, myo-inositol and carbon source have been scarcely evaluated for date palm organogenesis (Al-Khateeb 2008a; Mazri 2013, 2014; Mazri and Meziani 2013).

Major mineral salts (macronutrients) are inorganic elements needed in relatively large amounts for healthy and vigorous growth of plants (George and de Klerk 2008). Carbohydrates are essential elements for in vitro propagation of plants (Thorpe et al. 2008). They are employed as a source of energy and as an osmotic agent (Thorpe et al. 2008), and to replace the carbon which is normally fixed by plants from the atmosphere by photosynthesis (George and de Klerk 2008). The mineral and carbohydrate requirements for shoot bud proliferation of date palm seem to depend on the genotype used. With regard to l-glutamine and myo-inositol, their effects on date palm organogenesis have never been evaluated. l-Glutamine is an amino acid known for promoting and maintaining cell function (Newsholme et al. 2003). It has been used to promote somatic embryo proliferation, maturation and germination of many plant species, including date palm (Encina et al. 2014; Kim and Moon 2007; Zouine and El Hadrami 2007; Zouine et al. 2005). Myo-inositol is a vitamin that promotes cell and protoplast division (Bellini et al. 1990; Kim et al. 2003; Kiviharju et al. 2005), as well as callus growth (Sepehr and Ghorbanli 2002).

The success of date palm organogenesis is hampered by some physiological disorders: hyperhydricity, a morphological anomaly in which explants are glassy, water soaked and translucent (Debergh et al. 1992); tissue browning, which occurs due to the high levels of caffeoylshikimic acids contained in date palm tissues (Loutfi and El Hadrami 2005); and precocious rooting of organogenic cultures, which decreases bud formation (Al-Khateeb 2008b). The composition of the culture medium affects the incidence of these physiological disorders. Therefore, it is necessary to take into account their occurrence during the optimization of the culture medium.

The purpose of this investigation was to optimize the concentrations of mineral salts, l-glutamine, myo-inositol and carbon source for a rapid and efficient shoot bud multiplication of the recalcitrant date palm cultivar Mejhoul and to examine their effects on the incidence of hyperhydricity, tissue browning and precocious rooting. Accordingly, a total of 70 different culture media were evaluated.

## Materials and methods

### Plant material and culture conditions

This study used organogenic cultures, comprising four buds each, and obtained from shoot tip explants of date palm cv. Mejhoul as described by Beauchesne et al. (1986) with some modifications in the disinfection process. Briefly, offshoots (3-year-old) were removed from adult trees. The outer leaves were detached then the shoot tip was extracted. The shoot tip was subjected to disinfection with 0.03 % solution of potassium permanganate (Sigma, Steinheim, Germany) in commercial liquid bleach (ACE, Industries Marocaines Modernes, Casablanca, Morocco) for 20 min followed by three rinses with sterile distilled water. The explants used for organogenic culture initiation were taken from the meristematic region and then cultured for 9 months on half-strength Murashige and Skoog (1962) medium (MS/2) supplemented with 30 g/L sucrose (Sigma, St. Louis, MO, USA), 0.2 g/L l-glutamine (Sigma, St. Louis, MO, USA), 0.1 g/L myo-inositol (Sigma, St. Louis, MO, USA), 2 g/L polyvinylpyrrolidone (Duchefa Biochemie, Haarlem, The Netherlands), 3 mg/L 2-naphthoxyacetic acid (NOA), 1 mg/L 1-naphthaleneacetic acid (NAA), 1 mg/L indole-3-acetic acid (IAA), 0.1 mg/L N6-[2-isopentenyl] adenine (2iP) and 6 g/L agar (Sigma, St. Louis, MO, USA). The pH of the culture medium was adjusted to 5.6 before autoclaving at 121 °C for 25 min at 103 kPa. The explants were maintained in darkness at 25 °C and transferred to fresh medium every 4 weeks.

During the multiplication phase (experiments 1, 2, 3 and 4), all media (Table 1) were supplemented with 0.2 mg/L NOA, 0.2 mg/L IAA, 0.4 mg/L 2iP and 0.4 mg/L kinetin as suggested by Meziani et al. (2015). In all experiments, media were supplemented with 1 g/L polyvinylpyrrolidone, gelled with 6 g/L agar and the pH was adjusted to 5.7 prior to autoclaving for 25 min at 121 °C at 103 kPa. For each experiment, two organogenic cultures were placed in a jar (12 cm in height, 6.5 cm in diameter) containing 40 ml of the culture medium and cultured for 3 months. The explants were maintained at 25 °C in a 16 h photoperiod with a light intensity of 13.5 µmol m^−2^ s^−1^ provided by white cool fluorescent tubes, with transfer to fresh medium every month.

**Table 1:** Mineral salts, l-glutamine, myo-inositol and carbon source concentrations tested during shoot bud proliferation of date palm cv. Mejhoul

Medium component	Concentration					
	Standard medium	Experiment 1	Experiment 2	Experiment 3	Experiment 4	Improved medium
NH_4_NO_3_ (mg/L)	825	550–1650^a^	825	825	825	825
KNO_3_ (mg/L)	950	633.3–1900^a^	1900	1900	1900	1900
CaCl_2_·2H_2_O (mg/L)	220	220	147–440^a^	220	220	220
MgSO_4_·7H_2_O (mg/L)	185	185	123–370^a^	370	370	370
KH_2_PO_4_ (mg/L)	85	85	57–170^a^	170	170	170
l-Glutamine (g/L)	0.25	0.25	0.25	0.5–2^a^	1	1
Myo-inositol (g/L)	0.25	0.25	0.25	0.5–2^a^	2	2
Sucrose (g/L)	–	–	–	–	10–50^a^	30
Sorbitol (g/L)	–	–	–	–	10–50^a^	–
Mannitol (g/L)	–	–	–	–	10–50^a^	–
Commercial granulated sugar (g/L)	30	30	30	30	10–50^a^	–

All media were supplemented with vitamins and minor salts of MS basal formulation, 0.2 mg/L NOA, 0.2 mg/L IAA, 0.4 mg/L 2iP, 0.4 mg/L kinetin, 1 g/L PVP and 6 g/L agar

^a^The concentration range tested in each experiment

### Experimental protocols

#### Experiment 1: effects of ammonium nitrate (NH_4_NO_3_) and potassium nitrate (KNO_3_) concentrations on shoot bud proliferation

Organogenic cultures of date palm cv. Mejhoul were cultured on media consisting of various concentrations of NH_4_NO_3_ (550, 825 or 1650 mg/L) and KNO_3_ (633.3, 950 or 1900 mg/L); as well as 220 mg/L calcium chloride dehydrate (CaCl_2_·2H_2_O), 185 mg/L magnesium sulfate heptahydrate (MgSO_4_·7H_2_O), 85 mg/L potassium dihydrogen phosphate (KH_2_PO_4_), 30 g/L commercial granulated sugar (Cosumar, Casablanca, Morocco), 0.25 g/L l-glutamine, 0.25 g/L myo-inositol, vitamins and minor salts of MS medium.

#### Experiment 2: effects of CaCl_2_·2H_2_O, MgSO_4_·7H_2_O and KH_2_PO_4_ on shoot bud proliferation

Organogenic cultures were cultured for 3 months on media containing 825 mg/L NH_4_NO_3_, 1900 mg/L KNO_3_ (these concentrations were chosen according to the results from experiment 1), 30 g/L commercial granulated sugar, 0.25 g/L l-glutamine, 0.25 g/L myo-inositol, vitamins and minor salts of MS medium, and supplemented with various concentrations of CaCl_2_·2H_2_O (147, 220 or 440 mg/L), KH_2_PO_4_ (57, 85 or 170 mg/L), and MgSO_4_·7H_2_O (123, 185 or 370 mg/L).

#### Experiment 3: effects of l-glutamine and myo-inositol on shoot bud proliferation

In this experiment, four concentrations (0.5, 1, 1.5 and 2 g/L) of l-glutamine and myo-inositol were evaluated. l-Glutamine and myo-inositol were supplemented to the culture medium that was containing 825 mg/L NH_4_NO_3_, 1900 mg/L KNO_3_, 220 mg/L CaCl_2_·2H_2_O, 170 mg/L KH_2_PO_4_, 370 mg/L MgSO_4_·7H_2_O (based on the results of experiments 1 and 2), 30 g/L commercial granulated sugar, vitamins and minor salts of MS medium.

#### Experiment 4: effects of carbon source type and concentration on shoot bud proliferation

According to the results from the above experiments, and to evaluate the effects of carbon sources on shoot bud multiplication, organogenic cultures were cultured on media containing 825 mg/L NH_4_NO_3_, 1900 mg/L KNO_3_, 220 mg/L CaCl_2_·2H_2_O, 170 mg/L KH_2_PO_4_, 370 mg/L MgSO_4_·7H_2_O, 1 g/L l-glutamine, 2 g/L myo-inositol, vitamins and minor salts of MS medium, and various concentrations (10, 20, 30, 40 or 50 g/L) of sucrose, sorbitol, mannitol (all purchased from Sigma, St. Louis, MO, USA) or commercial granulated sugar.

### Shoot growth and acclimatization

For shoot elongation and rooting, 4.5–6 cm-long shoots were isolated from the organogenic cultures that were on the medium containing 825 mg/L NH_4_NO_3_, 1900 mg/L KNO_3_, 220 mg/L CaCl_2_·2H_2_O, 170 mg/L KH_2_PO_4_, 370 mg/L MgSO_4_·7H_2_O, 1 g/L l-glutamine, 2 g/L myo-inositol, 30 g/L sucrose, vitamins and minor salts of MS medium as well as PGRs. The shoots were cultured for 3 months on this same medium at 25 °C and 16 h photoperiod (40 µmol m^−2^ s^−1^), but without PGR. After the elongation–rooting phase, well-rooted plantlets with three to four leaves were transferred to the glasshouse as described by Mazri and Meziani (2013). Briefly, the plantlets were removed from the culture medium. The roots were washed in tap water then soaked for 15 min in a fungicide solution (1 g/L Pelt 44 PM; Bayer CropScience, Bayer Maghreb SA, Casablanca, Morocco). The plantlets were then transferred to plastic bags with a mixture of peat–gravel (1:1) substrate. The bags were placed in a polyethylene tunnel for 2 weeks (98 % relative humidity). After this period, the polyethylene tunnel was gradually opened to adapt plantlets to glasshouse conditions (70 % relative humidity; 27 °C).

### Data collection and statistical analysis

During the multiplication phase, there were ten replicates of each treatment, and each replicate consisted of one jar containing two organogenic cultures. After 3 months of culture, the mean number of shoot buds per organogenic culture, the mean number and length of precociously formed roots, the intensity and percentage of hyperhydricity and tissue browning were recorded. During the elongation–rooting phase, the recorded data after 3 months were shoot length (cm) and the mean number and length (cm) of roots. After 4 months in the glasshouse, the percentage of plantlets survival was calculated.

Data were analyzed by ANOVA using a completely randomized design at 5 % significance level, and the comparison of means was performed with the Student–Newman–Keuls test at *P* ≤ 0.05 (SPSS v. 16.0, IBM, Chicago, IL, USA). Percentage data were arcsine transformed prior to analysis.

## Results

### Experiment 1: effects of NH_4_NO_3_ and KNO_3_ concentrations on shoot bud proliferation

**Fig. 1:**
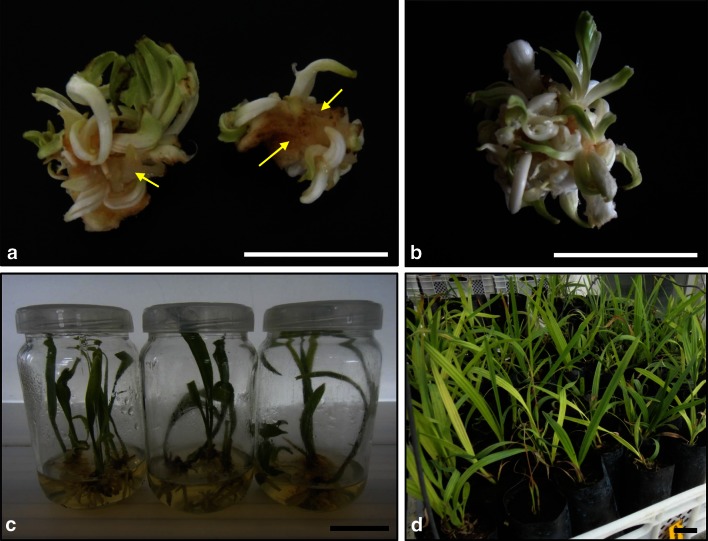
Shoot bud multiplication, elongation, rooting and acclimatization of date palm (*Phoenix dactylifera* L.) cv. Mejhoul. **a** High level of hyperhydricity in organogenic cultures after 1 month of culture on the medium containing 1650 mg/L NH_4_NO_3_, 1900 mg/L KNO_3_, 220 mg/L CaCl_2_·2H_2_O, 85 mg/L KH_2_PO_4_, 185 mg/L MgSO_4_·7H_2_O, 0.25 g/L l-glutamine, 0.25 g/L myo-inositol, 30 g/L commercial granulated sugar, vitamins and minor salts of MS medium.* Yellow arrows* indicate hyperhydricity. **b** Organogenic culture after 1 month of culture on the medium containing 825 mg/L NH_4_NO_3_, 1900 mg/L KNO_3_, 220 mg/L CaCl_2_·2H_2_O, 170 mg/L KH_2_PO_4_, 370 mg/L MgSO_4_·7H_2_O, 1 g/L l-glutamine, 2 g/L myo-inositol, 30 g/L sucrose, vitamins and minor salts of MS medium. **c** Shoots after 3 months on the PGR-free elongation–rooting medium. **d** Plantlet survival after 4 months in the glasshouse. *Bars* correspond to 3 cm

Various combinations of NH_4_NO_3_ and KNO_3_ were evaluated. The maximum capacity for shoot bud formation was observed on the medium containing 825 mg/L NH_4_NO_3_ and 1900 mg/L KNO_3,_ in which the average number of shoot buds per explant was 14.5. The use of NH_4_NO_3_ at the concentration of 1650 mg/L increased significantly (*P* ≤ 0.05) the incidence of hyperhydricity (Fig. 1a) and decreased the multiplication rate (Table 2). In addition, the buds formed on the medium containing 825 mg/L NH_4_NO_3_ and 1900 mg/L KNO_3_ were thicker and more vigorous. The different concentrations of NH_4_NO_3_ and KNO_3_ did not affect the frequencies of browning and precocious rooting.

**Table 2:** Effect of different concentrations of ammonium nitrate (NH_4_NO_3_) and potassium nitrate (KNO_3_) on shoot bud proliferation of date palm cv. Mejhoul

NH_4_NO_3_ (mg/L)	KNO_3_ (mg/L)	Average number of shoot buds	Frequency of hyperhydricity (%)	Intensity of hyperhydricity	Frequency of browning (%)	Intensity of browning	Frequency of precocious rooting (%)	Average number of roots
1650	1900	13.5 ± 3.4ab	65 ± 33.7a	++	30 ± 25.8a	+	25 ± 35.3a	1.6 ± 2.0a
1650	950	12.1 ± 4.1ab	65 ± 41.1a	++	20 ± 25.8a	+	35 ± 24.1a	1.3 ± 1.2a
1650	633.3	9.8 ± 3.4ab	60 ± 39.4a	++	20 ± 34.9a	+	35 ± 41.1a	1.2 ± 1.8a
825	1900	14.5 ± 3.0a	25 ± 35.3b	+	25 ± 26.3a	+	25 ± 26.3a	1.2 ± 1.5a
825^a^	950^a^	12.9 ± 1.2ab	20 ± 42.1b	+	30 ± 34.9a	+	30 ± 25.8a	1.1 ± 1.2a
825	633.3	9.1 ± 5.7b	25 ± 35.3b	+	25 ± 26.3a	+	20 ± 34.9a	1.1 ± 2.0a
550	1900	12.4 ± 3.7ab	20 ± 25.8b	+	20 ± 25.8a	+	35 ± 33.7a	1.1 ± 1.1a
550	950	9.3 ± 3.4b	20 ± 34.9b	+	30 ± 25.8a	+	25 ± 35.3a	1.2 ± 1.7a
550	633.3	8.9 ± 3.0b	20 ± 25.8b	+	30 ± 25.8a	+	20 ± 25.8a	1.2 ± 1.6a

Values are mean ± standard deviation of ten repetitions. Values with the same lowercase letter are not statistically different (*P* > 0.05)

Intensities of hyperhydricity, tissue browning and precocious rooting are visually estimated as +, ++ and +++ for low, moderate and high, respectively

^a^The standard concentrations habitually used for shoot bud proliferation

### Experiment 2: effects of CaCl_2_·2H_2_O, MgSO_4_·7H_2_O and KH_2_PO_4_ on shoot bud proliferation

After 3 months of culture, the concentrations of 220 mg/L CaCl_2_·2H_2_O, 370 mg/L MgSO_4_·7H_2_O and 170 mg/L KH_2_PO_4_ stimulated shoot bud proliferation, with an average of 15.3 shoot buds per explant (Table 3). Lower concentrations of CaCl_2_·2H_2_O, MgSO_4_·7H_2_O and KH_2_PO_4_ showed lower multiplication rates, while the concentration of 440 mg/L of CaCl_2_·2H_2_O did not increase the number of shoot buds per explant. On the other hand, it has been found that CaCl_2_·2H_2_O, MgSO_4_·7H_2_O and KH_2_PO_4_ concentrations do not affect hyperhydricity, tissue browning and precocious rooting of explants.

**Table 3:** Effect of different concentrations of calcium chloride dehydrate (CaCl_2_·2H_2_O), potassium dihydrogen phosphate (KH_2_PO_4_) and magnesium sulfate heptahydrate (MgSO_4_·7H_2_O) on shoot bud proliferation of date palm cv. Mejhoul

CaCl_2_·2H_2_O (mg/L)	MgSO_4_·7H_2_O (mg/L)	KH_2_PO_4_ (mg/L)	Average number of shoot buds	Frequency of hyperhydricity (%)	Intensity of hyperhydricity	Frequency of browning (%)	Intensity of browning	Frequency of precocious rooting (%)	Average number of roots
440	370	170	14.0 ± 1.8ab	25 ± 35.3a	+	30 ± 25.8a	+	25 ± 35.3a	0.9 ± 1.3a
440	370	85	14.3 ± 1.5ab	25 ± 26.3a	+	25 ± 35.3a	+	25 ± 35.3a	1.1 ± 1.7a
440	370	57	13.3 ± 2.0ab	20 ± 25.8a	+	30 ± 34.9a	+	30 ± 34.9a	0.7 ± 0.8a
440	185	170	13.9 ± 2.3ab	25 ± 35.3a	+	20 ± 25.8a	+	30 ± 42.1a	2.1 ± 3.0a
440	185	85	13.8 ± 2.3ab	20 ± 25.8a	+	25 ± 26.3a	+	25 ± 26.3a	1.6 ± 1.8a
440	185	57	13.5 ± 2.2ab	20 ± 25.8a	+	25 ± 26.3a	+	30 ± 25.8a	1.6 ± 1.7a
440	123	170	13.5 ± 2.8ab	25 ± 35.3a	+	20 ± 25.8a	+	20 ± 25.8a	1.0 ± 1.4a
440	123	85	13.1 ± 2.6b	25 ± 26.3a	+	25 ± 35.3a	+	20 ± 34.9a	1.3 ± 2.1a
440	123	57	13.2 ± 2.2b	20 ± 25.8a	+	20 ± 25.8a	+	25 ± 26.3a	1.0 ± 1.3a
220	370	170	15.3 ± 1.6a	15 ± 24.1a	+	25 ± 26.3a	+	30 ± 34.9a	1.4 ± 2.1a
220	370	85	13.9 ± 2.6ab	20 ± 25.8a	+	25 ± 35.3a	+	30 ± 25.8a	1.6 ± 1.7a
220	370	57	13.7 ± 2.2ab	20 ± 34.9a	+	20 ± 25.8a	+	25 ± 26.3a	1.0 ± 1.3a
220	185	170	14.2 ± 1.6ab	25 ± 26.3a	+	20 ± 25.8a	+	20 ± 25.8a	1.2 ± 1.3a
220	185	85	14.5 ± 3.0ab	25 ± 35.3a	+	25 ± 26.3a	+	25 ± 26.3a	1.2 ± 1.5a
220	185	57	13.3 ± 1.3ab	20 ± 25.8a	+	20 ± 25.8a	+	25 ± 26.3a	2.1 ± 2.2a
220	123	170	13.0 ± 2.3b	25 ± 26.3a	+	25 ± 26.3a	+	25 ± 26.3a	1.4 ± 1.7a
220	123	85	12.8 ± 2.2b	20 ± 25.8a	+	25 ± 26.3a	+	25 ± 26.3a	1.6 ± 1.7a
220	123	57	12.7 ± 2.7b	20 ± 25.8a	+	20 ± 25.8a	+	20 ± 25.8a	0.7 ± 1.0a
147	370	170	12.3 ± 1.3b	25 ± 26.3a	+	20 ± 25.8a	+	30 ± 25.8a	1.8 ± 1.7a
147	370	85	12.6 ± 2.0b	20 ± 25.8a	+	25 ± 26.3a	+	20 ± 25.8a	1.1 ± 1.9a
147	370	57	12.0 ± 1.1b	20 ± 42.1a	+	25 ± 26.3a	+	25 ± 26.3a	1.2 ± 1.3a
147	185	170	12.3 ± 2.1b	20 ± 25.8a	+	25 ± 26.3a	+	20 ± 25.8a	0.7 ± 1.0a
147	185	85	12.1 ± 1.5b	25 ± 35.3a	+	20 ± 25.8a	+	25 ± 26.3a	1.3 ± 1.3a
147	185	57	12.1 ± 2.8b	20 ± 25.8a	+	25 ± 26.3a	+	25 ± 26.3a	1.2 ± 1.2a
147	123	170	12.6 ± 2.8b	20 ± 25.8a	+	20 ± 25.8a	+	25 ± 35.3a	0.9 ± 1.2a
147	123	85	12.3 ± 1.5b	15 ± 24.1a	+	25 ± 26.3a	+	20 ± 25.8a	1.1 ± 1.5a
147	123	57	12.3 ± 2.8b	20 ± 25.8a	+	20 ± 25.8a	+	25 ± 26.3a	1.4 ± 1.8a

Values are mean ± standard deviation of ten repetitions. Values with the same lowercase letter are not statistically different (*P* > 0.05)

Intensities of hyperhydricity, tissue browning and precocious rooting are visually estimated as +, ++ and +++ for low, moderate and high, respectively

The findings of the first and second experiments demonstrated that the concentrations of the macro-elements employed do not affect tissue browning and precocious rooting of explants. The concentrations of 825 mg/L NH_4_NO_3_, 1900 mg/L KNO_3_, 220 mg/L CaCl_2_·2H_2_O, 370 mg/L MgSO_4_·7H_2_O and 170 mg/L KH_2_PO_4_ are the most appropriate for shoot bud multiplication of date palm cv. Mejhoul.

### Experiment 3: effects of l-glutamine and myo-inositol on shoot bud proliferation

The combination of 1 g/L l-glutamine and 2 g/L myo-inositol was found to be optimal for shoot bud proliferation. In fact, the average number of shoot buds per explant significantly increased to 17.6 with this combination, with no significant difference (*P* > 0.05) with the combination of 1.5 g/L l-glutamine and 2 g/L myo-inositol, in which an average of 17.5 shoot buds per explant was obtained (Table 4). The different concentrations of l-glutamine and myo-inositol did not show a significant effect on the incidence of hyperhydricity, tissue browning and precocious rooting.

**Table 4:** Effect of l-glutamine and myo-inositol concentrations on shoot bud proliferation of date palm cv. Mejhoul

l-Glutamine (g/L)	Myo-inositol (g/L)	Average number of shoot buds	Frequency of hyperhydricity (%)	Intensity of hyperhydricity	Frequency of browning (%)	Intensity of browning	Frequency of precocious rooting (%)	Average number of roots
0.5	0.5	15.4 ± 2.5b	20 ± 25.8a	+	25 ± 35.3a	+	25 ± 26.3a	1.1 ± 1.4a
0.5	1	15.9 ± 4.7ab	25 ± 42.4a	+	25 ± 26.3a	+	30 ± 42.1a	1.6 ± 2.1a
0.5	1.5	15.4 ± 2.5b	25 ± 26.3a	+	20 ± 42.1a	+	25 ± 26.3a	1.4 ± 1.7a
0.5	2	16.1 ± 3.2ab	20 ± 34.9a	+	25 ± 42.4a	+	30 ± 25.8a	1.3 ± 1.5a
1	0.5	16.1 ± 2.9ab	25 ± 26.3a	+	20 ± 34.9a	+	20 ± 25.8a	1.0 ± 1.6a
1	1	16.6 ± 1.8ab	25 ± 42.4a	+	20 ± 25.8a	+	20 ± 42.1a	1.1 ± 2.3a
1	1.5	17.1 ± 3.2ab	20 ± 42.1a	+	25 ± 26.3a	+	30 ± 25.8a	1.8 ± 2.4a
1	2	17.6 ± 3.4a	20 ± 25.8a	+	20 ± 25.8a	+	20 ± 34.9a	1.1 ± 1.9a
1.5	0.5	15.5 ± 2.7b	20 ± 25.8a	+	25 ± 42.4a	+	25 ± 35.3a	1.1 ± 1.5a
1.5	1	16.0 ± 3.1ab	25 ± 42.4a	+	20 ± 42.1a	+	20 ± 25.8a	1.2 ± 1.6a
1.5	1.5	16.9 ± 4.9ab	25 ± 35.3a	+	25 ± 35.3a	+	20 ± 34.9a	1.4 ± 2.3a
1.5	2	17.5 ± 3.6a	20 ± 34.9a	+	20 ± 42.1a	+	25 ± 35.3a	1.3 ± 1.7a
2	0.5	16.2 ± 3.3ab	25 ± 35.3a	+	25 ± 42.4a	+	25 ± 42.4a	1.2 ± 2.1a
2	1	16.8 ± 3.0ab	25 ± 26.3a	+	25 ± 35.3a	+	25 ± 35.3a	1.4 ± 2.2a
2	1.5	17.1 ± 2.6ab	20 ± 25.8a	+	20 ± 25.8a	+	25 ± 42.4a	1.3 ± 2.1a
2	2	17.2 ± 3.8ab	25 ± 35.3a	+	30 ± 42.1a	+	20 ± 34.9a	1.5 ± 2.4a

Values are mean ± standard deviation of ten repetitions. Values with the same lowercase letter are not statistically different (*P* > 0.05)

Intensities of hyperhydricity, tissue browning and precocious rooting are visually estimated as +, ++ and +++ for low, moderate and high, respectively

### Experiment 4: effects of carbon source type and concentration on shoot bud proliferation

After 3 months of culture, an average of 18.7 shoot buds per explant was obtained on the medium supplemented with 30 g/L sucrose. The use of sorbitol, mannitol and commercial granulated sugar at the same concentration showed lower multiplication rates (17.6–17.9 shoot buds per explant; Table 5). Higher sucrose concentrations did not increase the number of shoot buds, while lower concentrations produced lower number of shoot buds per explant (13.9–17.1). The buds produced on media supplemented with sucrose (Fig. 1b) were morphologically superior to those obtained on media supplemented with sorbitol, mannitol and commercial granulated sugar.

**Table 5:** Effect of carbon source type and concentration on shoot bud proliferation of date palm cv. Mejhoul

Carbon source type and concentration (g/L)				Average number of shoot buds	Frequency of hyperhydricity (%)	Intensity of hyperhydricity	Frequency of browning (%)	Intensity of browning	Frequency of precocious rooting (%)	Average number of roots
Sucrose	Sorbitol	Mannitol	Commercial granulated sugar							
10	–	–	–	13.9 ± 1.5bcde	20 ± 25.8a	+	20 ± 25.8a	+	20 ± 25.8a	0.6 ± 0.8a
20	–	–	–	17.1 ± 2.6abcd	20 ± 25.8a	+	20 ± 25.8a	+	20 ± 25.8a	0.8 ± 1.0a
30	–	–	–	18.7 ± 3.5a	20 ± 25.8a	+	20 ± 25.8a	+	25 ± 35.3a	1.1 ± 1.5a
40	–	–	–	18.4 ± 2.8a	30 ± 34.9a	++	25 ± 35.3a	+	30 ± 34.9a	1.5 ± 1.6a
50	–	–	–	18.6 ± 1.8a	30 ± 34.9a	++	30 ± 34.9a	+	25 ± 35.3a	1.3 ± 1.7a
–	10	–	–	13.7 ± 2.0cde	20 ± 25.8a	+	20 ± 25.8a	+	20 ± 25.8a	1.2 ± 1.7a
–	20	–	–	16.9 ± 3.4abcd	20 ± 25.8a	+	25 ± 26.3a	+	20 ± 25.8a	0.7 ± 0.9a
–	30	–	–	17.9 ± 2.0ab	25 ± 35.3a	+	20 ± 25.8a	+	20 ± 25.8a	1.0 ± 1.3a
–	40	–	–	18.0 ± 2.8ab	30 ± 25.8a	++	25 ± 26.3a	+	25 ± 26.3a	1.1 ± 1.2a
–	50	–	–	17.3 ± 1.8abc	30 ± 34.9a	++	30 ± 34.9a	+	30 ± 34.9a	1.2 ± 1.3a
–	–	10	–	13.4 ± 2.7de	20 ± 25.8a	+	20 ± 25.8a	+	15 ± 24.1a	1.2 ± 1.9a
–	–	20	–	16.3 ± 2.7abcde	25 ± 26.3a	+	25 ± 35.3a	+	20 ± 25.8a	1.2 ± 1.6a
–	–	30	–	17.8 ± 3.5abc	20 ± 25.8a	+	25 ± 35.3a	+	25 ± 35.3a	1.4 ± 1.9a
–	–	40	–	17.8 ± 3.1abc	30 ± 34.9a	+	30 ± 34.9a	+	30 ± 25.8a	2.4 ± 2.1a
–	–	50	–	17.6 ± 2.5abc	30 ± 25.8a	++	30 ± 25.8a	+	20 ± 25.8a	1.2 ± 1.6a
–	–	–	10	13.1 ± 2.1e	25 ± 35.3a	+	30 ± 42.1a	+	15 ± 24.1a	0.8 ± 1.4a
–	–	–	20	16.2 ± 3.0abcde	20 ± 25.8a	+	20 ± 34.9a	+	25 ± 26.3a	1.5 ± 1.9a
–	–	–	30	17.6 ± 3.4abc	20 ± 25.8a	+	20 ± 25.8a	+	20 ± 34.9a	1.1 ± 1.9a
–	–	–	40	17.1 ± 2.5abcd	25 ± 26.3a	+	30 ± 34.9a	+	30 ± 34.9a	1.8 ± 2.0a
–	–	–	50	17.4 ± 2.5abc	25 ± 35.3a	+	30 ± 42.1a	+	25 ± 26.3a	2.0 ± 2.1a

Values are mean ± standard deviation of ten repetitions. Values with the same lowercase letter are not statistically different (*P* > 0.05)

Intensities of hyperhydricity, tissue browning and precocious rooting are visually estimated as +, ++ and +++ for low, moderate and high, respectively

### Comparison of standard and improved media

The final improved medium for shoot bud multiplication of date palm cv. Mejhoul contained 825 mg/L NH_4_NO_3_, 1900 mg/L KNO_3_, 220 mg/L CaCl_2_·2H_2_O, 170 mg/L KH_2_PO_4_, 370 mg/L MgSO_4_·7H_2_O, 1 g/L l-glutamine, 2 g/L myo-inositol and 30 g/L sucrose. The average number of shoot buds produced in the improved medium was 18.7, while it was 12.9 in the standard medium (Table 6). The difference was statistically significant (*P* < 0.05) and the increase gained was 44.96 %.

**Table 6:** Comparison of shoot bud proliferation of date palm cv. Mejhoul when grown in standard and improved culture media

	Average number of shoot buds	Frequency of hyperhydricity (%)	Intensity of hyperhydricity	Frequency of browning (%)	Intensity of browning	Frequency of precocious rooting (%)	Average number of roots
Standard medium	12.9 ± 1.2a	20 ± 42.1a	+	30 ± 34.9a	+	30 ± 25.8a	1.1 ± 1.2a
Improved medium	18.7 ± 3.5b	20 ± 25.8a	+	20 ± 25.8a	+	25 ± 35.3a	1.1 ± 1.5a

Values are mean ± standard deviation of ten repetitions. Values with the same lowercase letter are not statistically different (*P* > 0.05)

Intensities of hyperhydricity, tissue browning and precocious rooting are visually estimated as +, ++ and +++ for low, moderate and high, respectively

### Shoot growth, rooting and acclimatization

Shoot elongation and rooting (Fig. 1c) were achieved on a PGR-free medium consisting of 825 mg/L NH_4_NO_3_, 1900 mg/L KNO_3_, 220 mg/L CaCl_2_·2H_2_O, 170 mg/L KH_2_PO_4_, 370 mg/L MgSO_4_·7H_2_O, 1 g/L l-glutamine, 2 g/L myo-inositol and 30 g/L sucrose. After 3 months of culture, the average length of shoots was 13.9 cm, the average number of roots per shoot was 4.3 while root length was 4.2 cm, and an interesting survival rate of plantlets of 90 % was obtained after 4 months in the glasshouse (Fig. 1d).

## Discussion

In date palm, Mejhoul is one of the most recalcitrant cultivars in tissue culture. In view of developing an efficient regeneration protocol for this cultivar, the most appropriate nutritional requirements for shoot bud multiplication were determined. The concentration of mineral salts strongly affected shoot bud multiplication of date palm cv. Mejhoul. Similar results have been observed in other plant species such as *Rubus idaeus* L. (Poothonga and Reed 2014), *Billbergia zebrina* (Martins et al. 2015) and some *Pyrus* genotypes (Reed et al. 2013). Mineral requirements for date palm-cultured tissue seem to vary among cultivars (Mazri and Meziani 2015). Generally, MS and MS/2 basal formulations have been widely used for date palm shoot bud multiplication. For example, in date palm cvs. Maktoom, Dhakki and Hillawi, MS medium was used (Khierallah and Bader 2007; Khan and Bi Bi 2012; Al-Mayahi 2014), while Mazri  2015 used MS/2 medium for cv. Boufeggous. In a previous study on cv. Najda, the effect of six basal formulations was evaluated and it has been found that MS/2 medium is the most appropriate for shoot bud multiplication (Mazri and Meziani 2013). In cv. 16-bis, MS medium showed higher multiplication efficiency than woody plant medium and Nitsch medium (Mazri 2013). In the present work, the combination of 825 mg/L NH_4_NO_3_, 1900 mg/L KNO_3_, 220 mg/L CaCl_2_·2H_2_O, 170 mg/L KH_2_PO_4_ and 370 mg/L MgSO_4_·7H_2_O considerably stimulated the formation of adventitious buds. It has been found also that hyperhydricity has increased at high concentrations of NH_4_NO_3_ in the medium. This confirms previous statements by Al-Khateeb (2008b) and Abahmane (2011) who reported that high levels of ammonium ions increase the incidence of hyperhydricity. On the other hand, the different levels of mineral salts evaluated did not affect the incidence of tissue browning and precocious rooting.

The addition of l-glutamine and myo-inositol at the concentrations of 1 and 2 g/L, respectively, significantly enhanced the proliferation of adventitious buds. l-Glutamine is an amino acid known for promoting and maintaining cell function (Newsholme et al. 2003). It is involved in animal cell division (Diestel et al. 2007) and is known to have anti-inflammatory properties (Jain and Khanna 1981), induce weight loss in overweight and obese human adults (Zambom de Souza et al. 2015) and facilitate genetic transformation of some plant species (Kumar et al. 2013). With regard to micropropagation, l-glutamine has been used to stimulate somatic embryo maturation and germination of many plant species such as *Picea mariana* (Khlifi and Tremblay 1995), *Pinus strobus* (Garin et al. 2000), *Larix leptolepis* (Kim and Moon 2007) and *Persea americana* (Encina et al. 2014). In date palm tissue culture, studies on the effects of l-glutamine are scarce. Zouine et al. (2005) and Zouine and El Hadrami (2007) reported that l-glutamine improves the proliferation of somatic embryos of cvs. Jihel and Bousthami Noir. The results of the present study indicate that l-glutamine improves also the proliferation of date palm shoot buds. In fact, increasing the concentration of l-glutamine to 1 g/L significantly increased the number of shoot buds per explant.

Myo-inositol is a vitamin that stimulates cell division and an osmotic stabilizer for sustaining cell division (Kim et al. 2003; Kiviharju et al. 2005). Myo-inositol has been used to promote callus growth (Sepehr and Ghorbanli 2002) and protoplast division (Bellini et al. 1990). To date, the effect of myo-inositol on date palm micropropagation has not been investigated. Interestingly, it has been found that myo-inositol promotes shoot bud proliferation of date palm cv. Mejhoul.

With regard to carbon source type and concentration, it has been found that 30 g/L sucrose is the most appropriate for shoot bud multiplication. Lower or higher concentrations of sucrose did not improve shoot bud proliferation. This confirms the findings of Taha et al. (2001), who found that 30 g/L sucrose is more effective than 10, 20 or 40 g/L for the multiplication of shoot buds of date palm cv. Zaghlool. However, in date palm cv. 16-bis, it has been found that the concentration of 20 g/L sucrose is the most appropriate for shoot bud multiplication (Mazri 2014). In cv. Khanezi, sugar type (sucrose, glucose, fructose and maltose) did not show a significant difference in bud formation; however, the concentrations of 30 and 60 g/L were the most appropriate in comparison with higher concentrations (Al-Khateeb 2008a). This shows that sugar requirement may differ among genotypes. In the present study, sorbitol, mannitol and commercial granulated sugar appeared to be less effective than sucrose in proliferating shoot buds of date palm cv. Mejhoul. This might be due to the different nutritional and osmotic potentials of these carbon sources, and their different effects on cell growth, differentiation and morphogenesis (Neto and Otoni 2003; Yaseen et al. 2013).

Shoot elongation and rooting were achieved on PGR-free medium as it was previously reported in other date palm cultivars (Mazri 2014, 2015). In fact, it has been found that shoot elongation and rooting on PGR-free media significantly increases the survival rate during the acclimatization phase (Mazri and Meziani 2013). In the present study, the survival rate observed after acclimatization was 90 %. Generally speaking, high survival rates were reported in date palm organogenesis (Khan and Bi Bi 2012; Khierallah and Bader 2007; Mazri 2015; Meziani et al. 2015).

In conclusion, mineral salts, l-glutamine, myo-inositol, as well as carbon source type and concentration significantly affect shoot bud proliferation of date palm cv. Mejhoul. The optimized culture medium will be useful for the rapid and large-scale propagation of this recalcitrant and endangered date palm cultivar. The effect of some natural compounds is currently being investigated to reduce tissue browning during the initiation and multiplication of adventitious buds of cv. Mejhoul.

## Abbreviations

CaCl_2_**·**2H_2_O: Calcium chloride dehydrate
KH_2_PO_4_: Potassium dihydrogen phosphate
KNO_3_: Potassium nitrate
MgSO_4_**·**7H_2_O: Magnesium sulfate heptahydrate
MS: Murashige and Skoog
NH_4_NO_3_: Ammonium nitrate
PGR: Plant growth regulator
